# One-Minute Room-Temperature Transfer-Free Production of Mono- and Few-Layer Polycrystalline Graphene on Various Substrates

**DOI:** 10.1038/srep19313

**Published:** 2016-01-14

**Authors:** Shenglin Jiang, Yike Zeng, Wenli Zhou, Xiangshui Miao, Yan Yu

**Affiliations:** 1 School of Optical and Electronic Information, Huazhong University of Science and Technology, Wuhan, Hubei province, 430074, PR China

## Abstract

Graphene deposited on various substrates has attracted the attention of the scientific and technical communities for use in a wide range of applications. Graphene on substrates is commonly produced by two types of methods, namely, methods that require a transfer step and transfer-free methods. Compared with methods that require a transfer step, transfer-free methods have a simpler procedure and a lower cost. Thus, transfer-free methods have considerable potential to meet the industrial and commercial demands of production methods. However, some limitations of the current transfer-free methods must be overcome, such as the high temperatures encountered during production, the relatively long manufacturing times, incompatibilities for both rigid and flexible substrates, and an inability to extend the process to other two-dimensional (2-D) atomic crystals. In this work, a room-temperature rubbing method is developed for the rapid transfer-free production of defect-free polycrystalline graphene on rigid and flexible substrates. Starting with inexpensive commercially obtained graphite powder, mono- and few-layer graphene can be fabricated directly on various substrates, with an average production time of less than one minute (from raw graphite to graphene on the substrate). Importantly, this method can be extended to other 2-D atomic crystals.

Graphene has attracted intense scientific and technical interest during the past decade, as its extraordinary properties make this material suitable for a wide range of applications, including electronics, photonics, energy, and mechanics^1^,^2^,^3^,^4^. There are two major forms of grapheme used in various applications^3^,^4^: graphene sheets, which mainly used for bulk applications, such as composites, coatings, inks, and energy storage^5^,^6^,^7^,^8^,^9^,^10^, and graphene films that are on substrates, which are mainly used for electronic and optoelectronic functions^8^,^9^,^10^,^11^,^12^,^13^,^14^,^15^,^16^. Graphene films on substrates are commonly produced by two types of methods^8^,^9^,^10^,^11^,^12^,^13^,^14^,^15^,^16^: methods that require a transfer step or transfer-free methods. Compared with the methods that require transferring, transfer-free methods have a simpler procedure and lower cost^8^,^9^,^10^,^11^,^12^,^13^,^14^,^15^,^16^. Thus, they are likely to meet industrial and commercial demands for production methods, such as reducing costs, simplifying procedures, and making the procedures environmentally friendly^4^,^17^,^18^,^19^,^20^.

In recent years, transfer-free methods based on different synthesis have been explored, including chemical vapour deposition (CVD) growth with metal catalysts, such as Cu, Ni, Ga, etc.^21^,^22^,^23^,^24^,^25^,^26^, metal-catalyst-free CVD growth^27^,^28^,^29^,^30^,^31^,^32^,^33^,^34^,^35^,^36^, and Ni-assisted carbon diffusion synthesis^21^,^37^,^38^,^39^,^40^,^41^. However, some of the constraints of the current transfer-free methods must be overcome^21^,^22^,^23^,^24^,^25^,^26^,^27^,^28^,^29^,^30^,^31^,^32^,^33^,^34^,^35^,^36^,^37^,^38^,^39^,^40^,^41^. Specifically, (1) the procedures typically require high temperatures (from several hundred degrees to higher than 1,000 °C), (2) the production times are relatively long (often hours), (3) a certain method of growth is unsuitable for both rigid (e.g., Si and SiO_2_) and flexible (e.g., various polymers) substrates, and (4) the methods are difficult to extend to other two-dimensional (2-D) atomic crystals.

Herein, a room temperature rubbing method is developed for the rapid transfer-free production of defect-free polycrystalline graphene on various types of substrates (including both rigid and flexible substrates). Starting with low-cost commercially available graphite powders, mono- and few-layer graphene were directly fabricated with an average production time of less than one minute (from raw graphite to graphene on substrates). Importantly, this rubbing method can be extended to other 2-D atomic crystals. Thus, this process has great potential to meet a number of industrial and commercial demands: it is more likely to produce polycrystalline films than the expensive single-crystalline films^2^,^42^,^43^, it uses low-cost raw materials^5^,^18^,^19^, the production temperature^22^,^38^ and time^27^,^37^ are reduced, it is conducive to simplified^19^,^37^ and environmentally friendly processing^17^,^18^, and it can be adapted for use with different 2-D atomic crystals^1^,^2^,^5^,^7^,^11^,^44^,^45^ and substrates^28^,^35^,^37^.

This rubbing method is an important improvement over our previous methods^46^,^47^. Compared with our previous works, critical technical updates have been made: (1) the previous “smooth rubbing” step is replaced by an innovative “double-smooth-rubbing” step, (2) the previous “soft-contact-rubbing” step is replaced by a new “repeated-production” step that can be repeatedly operated, (3) a “restorative-rubbing” step is proposed and added to the procedures. Thus, the functionalities of the rubbing method are greatly enhanced by these technical updates in the following ways: (1) the technique is suitable for both rigid (e.g., Si and SiO_2_) and flexible (e.g., various polymers) substrates instead of only being suitable for flexible substrates; (2) both mono and few-layer graphene can be produced instead of just few-layer graphene; (3) the average time from raw graphite powders to final graphene films is reduced from approximately 10 minutes to less than one minute; (4) the procedures are more environmentally friendly because none of the auxiliary materials are discarded; and (5) importantly, the advantages of the previous rubbing method are maintained, including room temperature and low-cost production, together with the capability of being extended to other 2-D atomic crystals.

## Results

### Production of Graphene on Substrates

**Figure 1:**
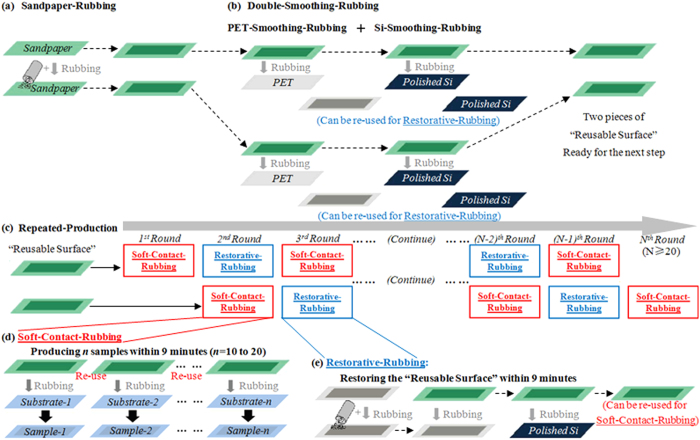
Diagram of the production steps: (a) Sandpaper-Rubbing Step. (**b**) Double-Smoothing-Rubbing Step. (**c**) Repeated-Production Step. (**d**) Soft-Contact-Rubbing procedure during Repeated-Production Step. (**e**) Restorative-Rubbing procedure during Repeated Producing Step.

Figure 1 presents a schematic of our production method. (1) The sandpaper-rubbing step (Fig. 1a) generates graphite sheets that are attached to sandpaper. (2) The double-smooth-rubbing step (Fig. 1b) creates reusable surfaces. (3) The repeated-production step (Fig. 1c) produces graphene samples on different substrates. (4) The soft-contact-rubbing step (Fig. 1d) is a sub-step of the repeated-production step (see the red shadow boxes in Fig. 1c), and its function is fabricating graphene on different substrates. (5) The restorative-rubbing step (Fig. 1e) is the other sub-step of the repeated-production step (see the blue shadow boxes in Fig. 1c), and its function is restoring the reusable surfaces for future use.

All of the auxiliary materials, including the poly(ethylene terephthalate) (PET) films, the polished silicon (Si) wafers, and, most importantly, the reusable surfaces that are built on the polishing sandpapers, are reusable. The surface morphologies of the auxiliary materials are given in Figures S1 and S2.

The reusability of the auxiliary materials means that we only needed to complete the sandpaper-rubbing (see Fig. 1a) and double-smooth-rubbing steps (see Fig. 1b) once to build two reusable surfaces, which can then be further used repeatedly for sample production (see Fig. 1c). During a single round of production, one piece of reusable surface was used to produce graphene on a certain type of substrate by the soft-contact-rubbing step (marked in the red shadow boxes in Fig. 1c and shown in detail in Fig. 1d), whereas the other piece of reusable surface could be utilized for the restorative-rubbing step (marked in the blue shadow boxes in Fig. 1c and shown in detail in Fig. 1e). Such a round of production only lasts for approximately 9 min (including time intervals between operations) but could produce 10 to 20 samples (see Fig. 1d). Immediately after the above round, production could resume by simply changing the roles of the two reusable surfaces (see Fig. 1c).

According to our experiments, these production cycles could continue for more than 20 rounds (commonly 22 to 34 rounds). If we count the rounds as 20, then the total time is 10 + 9 × 20 = 190 min, and the total sample number is (10 to 20) × 20 = 200 to 400. Therefore,1 sample only takes 0.475 to 0.95 min, in other words, within one minute.

### Characterization Results of Graphene on Substrates

**Figure 2:**
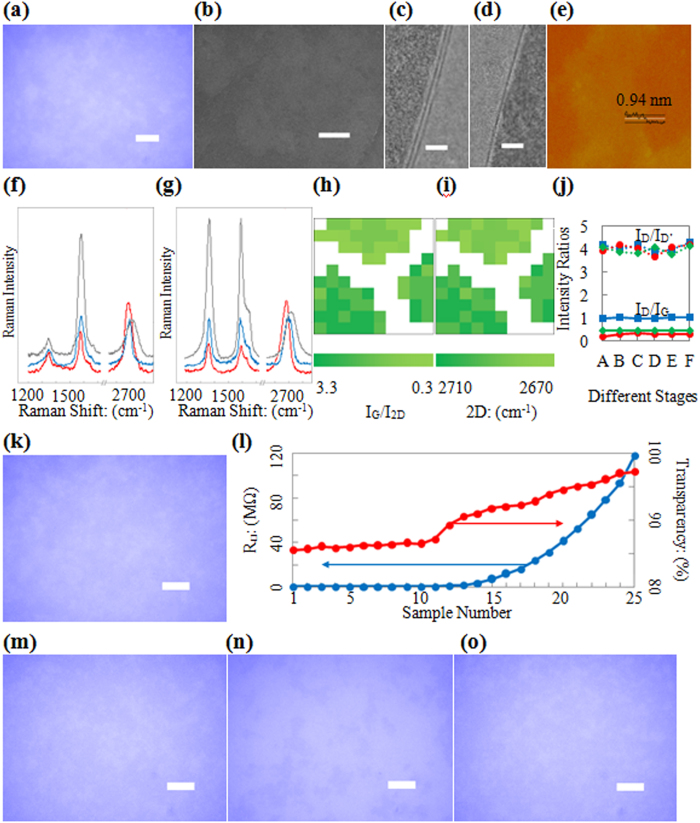
Graphene on different substrates. (**a**) Optical microscopy image of graphene on SiO_2_ substrate (scale bar, 10 μm). (**b**) SEM image of graphene on SiO_2_ substrate (scale bar, 1 μm). (**c**) TEM image of a double-layer graphene (scale bar, 2 nm). (**d**) TEM image of a mono-layer graphene (scale bar, 2 nm). (**e**) AFM image of graphene on Si substrate (area: 10 μm × 10 μm). (**f**) Raman results of graphenes (red and blue lines) on Si substrate and corresponding raw graphite (grey line). (**g**) Raman results of graphenes (red and blue lines) on Si substrate and corresponding raw graphite (grey line). (**h**) Raman mapping image of I_G_/I_2D_ intensity ratio (area: 10 μm × 10 μm). Raman laser whose spot size is 1 μm × 1 μm is moved by 1 μm. (**i**) Raman mapping image of 2D peak position of the same region as Fig. 2h (area: 10 μm × 10 μm). (**j**) Additional Raman results for defects study of graphene. (**k**) Optical microscopy image of graphene on PET substrate (scale bar, 10 μm). (**l**) Properties of samples during one production round. (**m**) Optical microscopy image of the 10^th^ sample during one production round (scale bar, 10 μm). (**n**) Optical microscopy image of the 30^th^ sample during one production round (scale bar, 10 μm). (**o**) Optical microscopy image of the 1^st^ sample during the 20^th^ production round (scale bar, 10 μm).

In this work, we chose a Si wafer and quartz glass (SiO_2_) as rigid substrates, whereas PET, polyethylene (PE) and polyurethane (PU) were employed as flexible substrates. All of the chosen substrates have smooth surfaces (surface roughness within 1 nm). The results of graphene on different substrates are presented in Fig. 2.

The results of graphene on SiO_2_ substrates are given in Fig. 2a–d. Figure 2a is an optical image of uniformly distributed graphene flakes that are tens of micrometres in size. Figure 2b presents a scanning electron microscope (SEM) image of the graphene on a SiO_2_ substrate. Figure 2c,d show transmission electron microscope (TEM) images of monolayer and bilayer graphene which were peeled off of the SiO_2_ substrate and transferred onto a TEM grid.

The results of graphene on Si substrates are given in Fig. 2e–j. Figure 2e contains atomic force microscope (AFM) images of graphene on the Si substrate. The thicknesses of the graphene flake shown in Fig. 2e are approximately 0.94 nm. The appearance of our AFM images is discussed in the following by additional SEM results and comparison of a AFM result of liquid exfoliated graphene flakes. In our SEM image, the irregular shapes are also exist, thus we could test the element purity of such irregular shape area. In this work, EDS was used to detect the element purity of graphene on our sample (see Figure S5a). In order to eliminate the influence of elements such as Oxygen, Nitrogen, *etc*., for EDS, we chose PE as polymer substrate for this test rather than PET, for the reason that only Oxygen and Hydrogen are contained in PE and Hydrogen can’t be detect by EDS. In this condition, if the elements detecting result in the chosen area (marked by red box in Figure S5a) shows no other element except Carbon (C), such area could be considered to be graphene (the element containing tiny impurities from raw graphite powders). In this sense, the results in the following figure did demonstrate the irregular shapes of graphene on our sample. Also, liquid exfoliated graphite nanosheets and graphene flakes (using our graphite powder from Aladdin Industrial Inc, as starting materials, and prepared in pure water, similar as Ref. S1) were also deposited on Si substrate by dip coating method. The sample was baked in oven for 10 hours at 70 ^o^C, to evaporate the water residues as completely as possible. Figure S5b gives the AFM image of a graphene flake which was found during AFM scanning procedure. And such image is similar to the AFM image of our graphene on Si substrate.

From the 514 nm Raman spectra in Fig. 2f,g, a clear red shift of the 2D peak can be observed in both the top and bottom. The 2D peak for raw graphite powder appears at approximately 2720 cm^−1^ and then shifts to approximately 2690 cm^−1^ for few-layer graphene and 2680 cm^−1^ for monolayer graphene. In addition, the G/2D intensity ratio decreased significantly from the graphite powders to the monolayer graphene, which is consistent with the literature^1^,^5^,^48^. Figure 2h,i display the Raman mapping images that correspond to an area containing a monolayer graphene flake (the top one) and two few-layer graphene flakes (the below two). Analysis of the number of layers were performed by considering both the Intensity ratio of I_G_/I_2D_ and the position of the 2D peak.

The results of graphene on flexible substrates are given in Fig. 2h,i. Figure 2h is the optical image of graphene on the PET substrate, and these graphene flakes is presented on a scale of tens of micrometres, similar to Fig. 2a.

### Defects and Purity of Graphene

The Raman D/D’ band intensity ratio and Raman D/G band intensity ratio were studied in detail to determine the level of defects in the graphene on the substrates. For comparison, three different commercial graphite powders were chosen because they have different Raman D/G band intensity ratios (see stage A in Fig. 2j); in other words, they exhibit different amounts of crystallinity (see SEM images in Figure S3). The Raman D/D’ band intensity ratios are shown in Fig. 2j (dotted lines) and remain fairly constant or the graphite powders (stage A in Fig. 2j),the graphite on the sandpapers’ surfaces (stage B represents the stage after sandpaper-rubbing, stage C represents the stage after PET-smooth-rubbing, stage D represents the stage after Si-smooth-rubbing, and stage E represents the stage after restorative rubbing) and the final graphene samples (stage F in Fig. 2j). All of the Raman D/D’ band intensity ratios are between 3.6 and 4.3. These results suggest that the defects of the graphene samples are boundary (or edge) defects that had already existed in the raw graphite powders^5^,^49^. The Raman D/G band intensity ratios show no clear changes once the value reaches approximately 1/3. These results reveal two important aspects. First, if the raw graphite powders have a relatively high crystallinity, i.e., a relatively low Raman D/G band intensity ratio of 0.19 (see the red solid line in Fig. 2j), then the rubbing procedures would likely decrease the crystallinity, which would result in an increase in the D/G ratio from 0.19 to 0.34(see the red solid line in Fig. 2j). Second, if the raw graphite powders have relatively low crystallinities with relatively high D/G ratios of 0.47 or 1.02 (see the green and blue solid lines in Fig. 2j), then the rubbing procedures would not further decrease the crystallinity, and the D/G ratios would exhibit no clear change (see the green and blue solid lines in Fig. 2j).

The above results imply that the observed defects are only boundary (or edge)-type defects^5^,^7^,^49^. Furthermore, such boundary (or edge) defects have their own advantages for certain applications^7^,^42^,^50^,^51^,^52^, such as humidity sensing, which will be discussed in part “Sample Properties and Applications”.

The elemental purity of the graphene is another important feature to characterize the quality of the produced graphene. In this work, energy dispersive spectroscopy (EDS) was used to detect the different elements present in our graphene sample. The result (Figure S4) reveals that aside from the Cu signal (from the TEM support), the sample contains carbon (C) of high purity.

### Cyclic Operability of the Method

Cyclic operability is one of the main advantages of the proposed method. There are two important issues: *(1)* How many samples can a reusable surface produce? *(2)* If possible, then how can the reusable surface be restored for further production?

Figure 2l–n provide an answer to the first question. During the initial 40 s of soft-contact-rubbing, one reusable surface can produce 10 samples with only small differences in the macro-performance (see Fig. 2l) and highly similar micro-morphologies (compare Fig. 2k,m). However, from the 11^th^ sample on, relatively large differences in the macro-performance (a considerably higher resistivity and higher transparency) and significant changes in the micro-morphology (compare Fig. 2k,n) are observed. Thus, for the 40 s of soft-contact-rubbing, we chose to produce 10 samples using one reusable surface.

For the second issue, it is possible to restore the depleted reusable surface via the restorative-rubbing step, and more than 20 restorations can be applied to a reusable surface. Figure 2m shows the 1^st^ sample of the 20^th^ production round by soft-contact-rubbing. This sample displays properties that are consistent with the first sample of the 1^st^ production round in terms of both the macro-performance (R_□_ = 573 Ω, T = 87.2%) and the micro-morphology (compare Fig. 2k,o).

The reusable surface can produce different numbers of samples for different soft-contact-rubbing times. Based on our experience, the number can be estimated as 400/(soft-contact-rubbing time). For example, for 40 s of soft-contact-rubbing, the number is 400/40 = 10, whereas the number of samples increases to 400/20 = 20 for 20 s.

### Controllability of the Method

During each rubbing step, there are three important parameters that impact the controllability: friction pressure (*P*), friction speed (*V*), and friction time (*t*). *t* was selected as the core parameter to be adjusted for the following reasons:*(1)* Under industrial conditions, *t* is easier to adjust and control than *P* and *V*. *(2)* Relatively higher *P* and *V* are favourable to improve the production efficiency; however, *P*·*V* has a direct relationship with the power requirements of the equipment, and such power is limited. Thus, only a relatively small adjustment range is possible for *P* and *V*. Consequently, *t* is the best parameter to describe the controllability and cyclic operability of the method.

**Figure 3:**
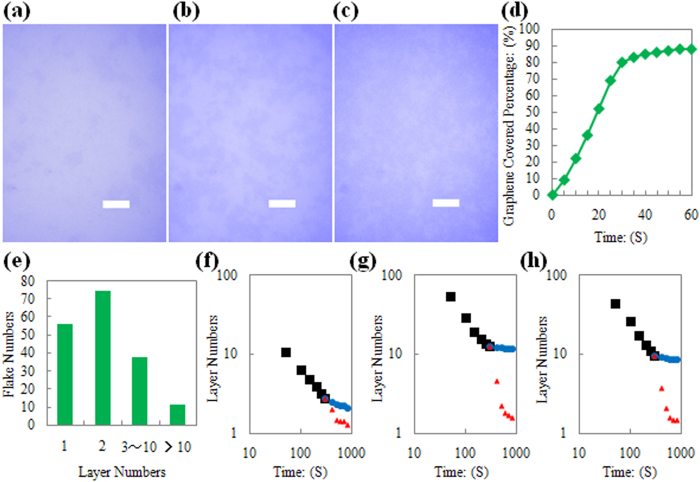
Controllability of the Method. (**a**) Optical image of graphenes on SiO_2_ substrates after 5 seconds soft contact rubbing (scale bar, 10 μm). (**b**) Optical image of graphenes on SiO_2_ substrate after 15 seconds soft contact rubbing (scale bar, 10 μm). (**c**) Optical image of graphenes on SiO_2_ substrate after 60 seconds soft contact rubbing (scale bar, 10 μm). (**d**) Graphene covered area percentage variation with soft contact rubbing time length. (**e**) Statistics results of graphene with certain layer numbers. (**f**) Layer number of graphene on PET substrates as a function of double smoothing rubbing conditions. (**g**) Layer number of graphene on SiO_2_ substrates as a function of double smoothing rubbing conditions. (**h**) Layer number of graphene on Si substrates as a function of double smoothing rubbing conditions.

The ability to adjust the area covered by graphene is an important metric for the controllability of the method. During the soft-contact-rubbing step, *t* has an important impact on the graphene covered area: when *t* is greater than 40 s, there is no discernible enlargement of the graphene-covered area (comparing Figs 2k and 3c), whereas a *t* of less than 40 s results in the apparent increase in the area covered by graphene (comparing Figs 3a,b and 2k). The variation in percent coverage with soft-contact-rubbing time is shown in Fig. 3d. Such controllability can be utilized for certain applications to produce graphene films with different properties, as discussed in the next Section.

Control over the number of graphene layers that are deposited is the other important parameter. The layer numbers were obtained from thickness results of AFM tests (details are listed in Supplementary Information). Figure 3e presents the total statistical results for the number of graphene layers on PET, Si and SiO_2_ substrates. Most of the graphene is monolayer or bilayer. Selectivity of the number of layers is mainly achieved by the double-smoothing-rubbing step. This step contains two consecutive smoothing operations (PET-smoothing-rubbing sub-step and Si-smoothing-rubbing sub-step): the initial rubbing uses PET as the smoothing material and is followed by smoothing with a polished Si wafer. The impacts of smooth-rubbing-time and smoothing materials (PET or Si) on the number of deposited graphene layers are compared in Fig. 3f–h (The error data are given in Tables S1-S9). It can be concluded that PET is suitable as the initial smoothing material (see the black points in Fig. 3f–h), while a polished Si wafer (red points in Fig. 3f–h) works better for additional smoothing (blue points in Fig. 3f–h).

### Sample Properties and Applications

**Figure 4:**
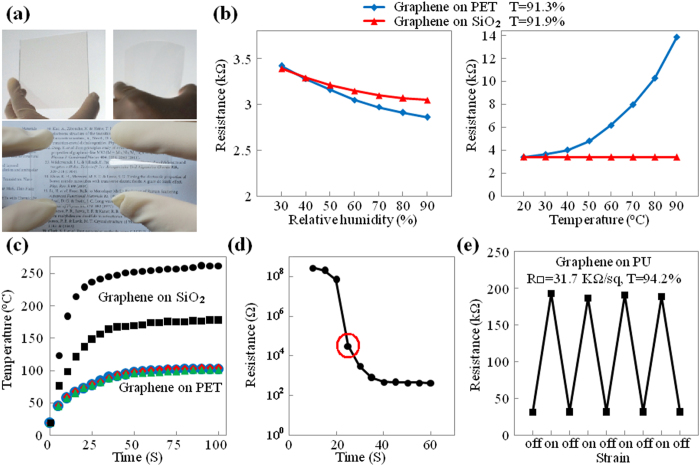
Sample Properties and Applications. (**a**) Photograph of graphene/PET and graphene/SiO_2_ samples with different sizes. (**b**) Changes in resistance as a function of the relative humidity (left part) and temperature (right part) of graphene/SiO_2_ sample (red line) and graphene/PET sample (blue line). (**c**) Transparent heating functions of graphene/SiO_2_ sample (black lines) and graphene/PET sample (colour lines). (**d**) Graphene/PU film’s surface resistivity as a function of soft-contact-rubbing time. (**e**) Strain sensing property of graphene/PU sample (with 25 seconds of soft-contact-rubbing time).

Graphene films on rigid and flexible substrates (see Fig. 4a) were used for applications, including humidity sensing, transparent heating, and strain sensing.

Polycrystalline grain boundaries can have a strong influence on the properties of graphene films, and also allow the preparation of materials with novel properties^7^,^42^,^50^,^51^,^52^. Here, the application of our polycrystalline graphene films as transparent humidity sensors was investigated. In this section, we used raw graphite powder with a relatively low degree of crystallinity (see the Raman results in Fig. 2h and the SEM image of the raw graphite powders in Figure S3a). SiO_2_ and PET were used as rigid and flexible substrates for the transparent humidity sensors and displayed high transparency (see Fig. 4a,b). After printing silver contacts, the properties of these humidity sensors were studied. The left part of Fig. 4b shows two curves of resistance, ΔR, changing as a function of relative humidity. The change in resistance rates was approximately 20% for relative humidity values from 30% to 90%, demonstrating the usefulness of this method for transparent humidity sensing. It is important to note that the rigid humidity sensor showed significantly better temperature stability than the flexible humidity sensor (see the right part of Fig. 4b). The reason for such a difference is that the temperature stability of SiO_2_ is much higher than PET (PET would be thermally expanded as temperature increases, thus increase the gaps between graphene flakes, leading to a increment of resistivity, while SiO_2_ would not be thermally expanded at such a temperature range, thus the resistivity would not be changed). These results indicate that the rigid humidity sensor can be used at a relatively high temperature, whereas the PET humidity sensor has the advantage of high flexibility during operation.

Figure 4c shows that graphene films on both rigid and flexible substrates could be used as transparent heaters: while the flexible heater is highly durable against mechanical ageing, the rigid heater shows a much higher operating temperature. It is worthy to note that for this application, we employed raw graphite powders with relatively high crystallinity (see Raman results in Fig. 2h and the SEM image of the raw graphite powders in Figure S3c).

Figure 4d,e show graphene films on stretchable PU substrates for strain sensing. This type of strain sensing is based on percolating networks of graphene flakes, and the sensing mechanism originates from strain-dependent changes to the film morphology, which was exploited to obtain films with a high strain gauge factor. From the results in Fig. 4d (film surface resistivity as a function of soft-contact-rubbing time), it is expected that the highest gauge factor might be obtained from samples that have properties near the percolation threshold, i.e., samples that have been subjected to soft-contact-rubbing for 20 to 40 s, and the final results (see Fig. 4e and Figure S6) indicated that the highest gauge factor was indeed obtained from a sample with 25 s of soft-contact-rubbing. The curves describing the change in resistance ΔR as a function of mechanical strain, Δε, demonstrate a strain gauge factor as high as 165 (calculated per the literature^53^), which reveals the applicability of this material as a transparent strain sensor.

The influence of the graphene sample size on its properties is crucial for industrial and commercial purposes. Notably, the results in Table S10 demonstrate a very high consistency between samples with different sizes (from 3 cm × 3 cm to 12 cm × 12 cm).

### Expanding the Method to other 2-D Atomic Crystals

In recent years, 2-D atomic crystals have been explored extensively, and examples of such crystals include those from hexagonal boron nitride (h-BN)^1^,^5^,^6^,^54^, transition metal dichalcogenides^1^,^5^,^6^,^54^,^55^,^56^,^57^, such as molybdenum disulfide (MoS_2_), tungsten disulfide (WS_2_), etc.

**Figure 5:**
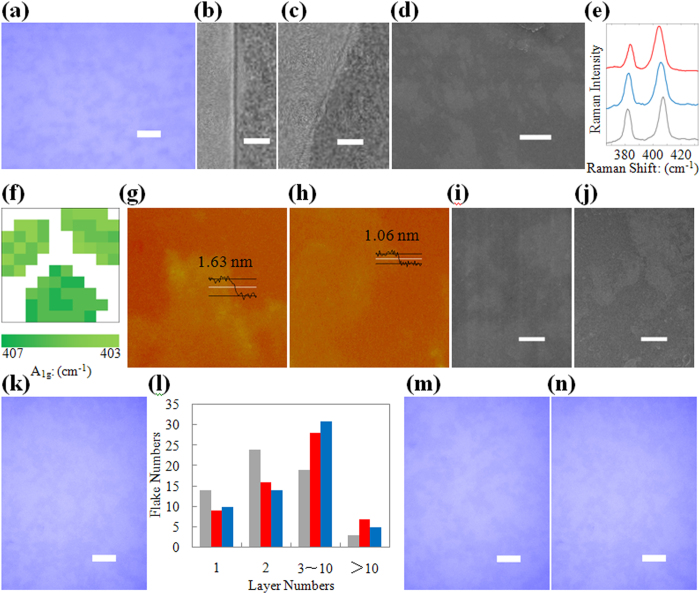
Other 2-D atomic crystals on different substrates. (**a**) Optical microscopy image of MoS_2_ atomic crystals on SiO_2_ substrate (scale bar, 10 μm). (**b**) TEM image of a mono-layer MoS_2_ atomic crystal (scale bar, 2 nm). (**c**) TEM image of a mono-layer h-BN atomic crystal (scale bar, 2 nm). (**d**) SEM image of WS_2_ atomic crystals on SiO_2_ substrate (scale bar, 1 μm). (**e**) Raman Results of MoS_2_ atomic crystals (red and blue lines) on Si substrate and correspongding raw MoS_2_ (grey line). (**f**) Raman mapping image of A_1g_ peak position (area: 10 μm × 10 μm). Raman laser whose spot size is 1 μm × 1 μm is moved by 1 μm. (**g**) AFM image of h-BN atomic crystal on Si substrate (area: 10 μm × 10 μm). (**h**) AFM image of MoS_2_ atomic crystal on Si substrate (area: 10 μm × 10 μm). (**i**) SEM image of h-BN atomic crystals on PET substrate (scale bar, 1 μm). (**j**) SEM image of MoS_2_ atomic crystals on PET substrate (scale bar, 1 μm). (**k**) Optical microscopy image of WS_2_ atomic crystals on PET substrate (scale bar, 10 μm). (**l**) Statistics results of h-BN (grey), MoS_2_ (red), WS_2_ (blue) atomic crystals with certain layer numbers. (**m**) Optical microscopy image of the 10^th^ MoS_2_ atomic crystals/SiO_2_ sample during one production round (scale bar, 10 μm). (**n**) Optical microscopy image of the 1^st^ WS_2_ atomic crystals/SiO_2_ sample during the 20^th^ production round (scale bar, 10 μm).

Figure 5 gives the results from three common 2-D atomic crystals (h-BN, MoS_2_ and WS_2_) produced on different substrates by the rubbing method contained within. SEM images of the raw materials are given in Figure S7.

The results showing 2-D atomic crystals on SiO_2_ substrates are given in Fig. 5a–d. Figure 5a is an optical image of MoS_2_ atomic crystals, which form a film with individual crystals on a scale of several micrometres. Fig. 5b,c show TEM images of monolayer MoS_2_ and monolayer h-BN, which were transferred from SiO_2_ to TEM grids. Figure 5d provides an SEM image of WS_2_ atomic crystals on a SiO_2_ substrate.

The results of 2-D atomic crystals on Si are given in Fig. 5e–h. Figure 5e shows that the 514 nm (*A*_*1g*_) peaks in the Raman spectra exhibit clear red shifts: approximately 2 cm^−1^ for few-layer MoS_2_ and approximately 5 cm^−1^ for monolayer MoS_2_. In addition, blue shifts of 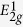 peaks are also visible, approximately 0.5 cm^−1^ and approximately 2 cm^−1^ for few-layer and monolayer MoS_2_, respectively. Such results were consistent with results from the literature on Raman spectra of bulk MoS_2_ and MoS_2_ atomic crystals^1^,^54^,^55^,^56^,^57^. Figure 5f presents Raman mapping images displaying an area that contains both mono-and few-layer MoS_2_ atomic crystal flakes. Figure 5g contains an AFM image of h-BN atomic crystals on a Si substrate. The thicknesses of the two tested h-BN atomic crystal in Fig. 5f was approximately 1.63 nm. Figure 5h displays AFM images of MoS_2_ atomic crystal on Si with thicknesses of approximately 1.06 nm.

The results of 2-D atomic crystals on flexible PET substrates are given in Fig. 5i–k. Figure 5I,j provide SEM images of h-BN and MoS_2_ atomic crystals on PET. Figure 5k is an optical image of WS_2_ on PET that presents atomic crystals on a scale of several micrometres, similar to the case of MoS_2_.

The controllability and cyclic operation of the method as applied towards 2-D atomic crystals are shown in Fig. 5l–n. Figure 5l gives the statistical results of the layer numbers for deposited h-BN, MoS_2_ and WS_2_ atomic crystals on Si, and just like graphene, most of these atomic crystals are monolayer and bilayer. The similarities between Fig. 5m,a demonstrate that one reusable surface can produce ten MoS_2_ atomic crystals/SiO_2_ sample. While the similarities of Fig. 5n,k demonstrate that the reusable surface can be restored and reused for 20 cycles with WS_2_ atomic crystals on PET.

## Discussion

The rubbing procedures involve the wear processing of the sandpaper surface, e.g., during the double-smoothing-rubbing step, the surface of the sandpaper is worn by PET and the polished Si wafer, while the soft-contact-rubbing step results in the sandpaper’s surface being worn by different substrates. Thus, classical friction and wear theories can be applied to explain the mechanisms behind the rubbing procedures.

According to the adhesive wear mechanism^58^, the wear rate (d’) is proportional to the pV factor, as described in Equation 1, where p is the apparent normal pressure, V is the sliding velocity, and d’ is the rate of wear depth (d/t) (mm/s) (t is the sliding time or duration).


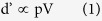


During the early stages, the rates of wear (d’) on sandpaper’s surface is proportional to pV. Because a certain pV value is already chosen, d’ is approximately constant at the beginning of the soft-contact-rubbing step. Thus, the amount of wear on the sandpaper’s surface increases with rubbing time (t), as does the amount of graphene transferred to the substrates.

According to Archard’s view^59^, the adhesive wear coefficient (K_ad_) can be considered proportional to the friction coefficient, as described in Equation 2.


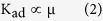


Thus, at a later stage of the soft-contact-rubbing, the rate of wear (d’) on the sandpaper’s surface is decreased, because graphene that was already transferred to the substrates would decrease the friction coefficient between the sandpaper and the substrates^60^. Thus, the transfer rate of graphene to the substrates decrease relative to the early stage.

**Figure 6:**
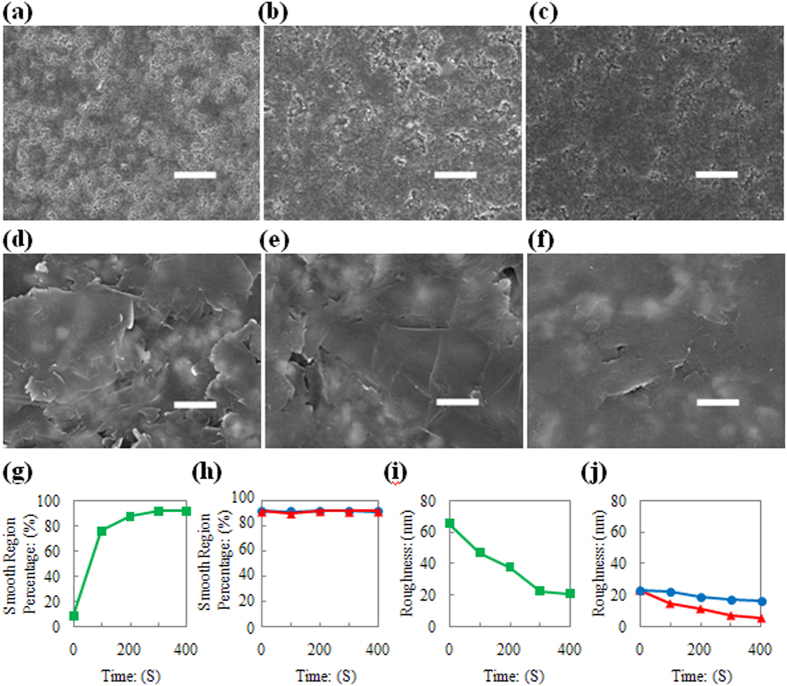
Mechanisms for layer number controllability. (**a**) SEM image of sandpaper’s surface after 100 seconds PET initial smoothing rubbing (scale bar, 20 μm). (**b**) SEM image of sandpaper’s surface after 300 seconds PET initial smoothing rubbing (scale bar, 20 μm). (**c**) SEM image of sandpaper’s surface after 300 seconds Si further smoothing rubbing (scale bar, 20 μm). (**d**) SEM image of smooth region on sandpaper’s surface, after 100 seconds PET initial smoothing rubbing (scale bar, 1 μm). (**e**) SEM image of smooth region on sandpaper’s surface, after 300 seconds PET initial smoothing rubbing (scale bar, 1 μm). (**f**) SEM image of smooth region on sandpaper’s surface, after 300 seconds Si further smoothing rubbing (scale bar, 1 μm). (**g**) Smooth area percentage on sandpaper’s surface as a function of PET initial smoothing rubbing time length. (**h**) Smooth area percentage on sandpaper’s surface as a function of further smoothing rubbing conditions and time length. (**i**) Smoothness of smooth region on sandpaper’s surface as a function of PET initial smoothing rubbing time length. (**j**) Smoothness of smooth region on sandpaper’s surface as a function of further smoothing rubbing conditions and time length.

Figure 6a,b clearly illustrate the important change in the sandpaper surface after the PET-smoothing-rubbing procedure. In this case, the area of the coarse region became smaller and the area of the smooth region became larger (see Fig. 6g). Furthermore, the smoothness of the smooth region improved (compare Fig. 6d,e, and see Fig. 6i). However, just using PET as a smoothing material is insufficient. After the early stage of double-smoothing-rubbing step, continued use of the PET as a smoothing material did not increase the smooth region area significantly (see blue points in Fig. 6h), and more importantly, it did not substantially decrease the roughness of the smooth region on the sandpaper’s surface (see blue points in Fig. 6j).

Therefore, a polished Si wafer is chosen because it exhibits a similar surface roughness to PET and has a much higher hardness. According to Rabinowicz’ view on wear^56^, the rates of wear (d’) on a smooth region of the sandpaper’s surface can be enhanced by a polished Si wafer. This leads to a more rapid reduction in the roughness of the smooth regions on the sandpaper’s surface (compare red and blue points in Fig. 6j). After the Si-smoothing-rubbing procedure, the ratio of coarse to smooth regions did not change significantly (compare Fig. 6b,c and see the red points in Fig. 6h). However, the smoothness of the smooth region is still improved (compare Fig. 6e,f and see the red points in Fig. 6j). Such changes to the sandpaper surface have an interesting parallel to the controllability of the number of graphene layers, as shown in Fig. 3f–h.

Three additional processing conditions (denoted as Conditions 1, 2 and 3) were carried out. Condition 1replaces the initial smoothing material (PET) in the double-smoothing-rubbing step with a polished Si wafer. Condition 2 replaces the second smoothing material (Si) in the double-smoothing-rubbing step with PET. Condition 3 changes the sequence of the double-smoothing-rubbing step (Si is the initial smoothing material, and PET is the second smoothing material).

**Figure 7:**
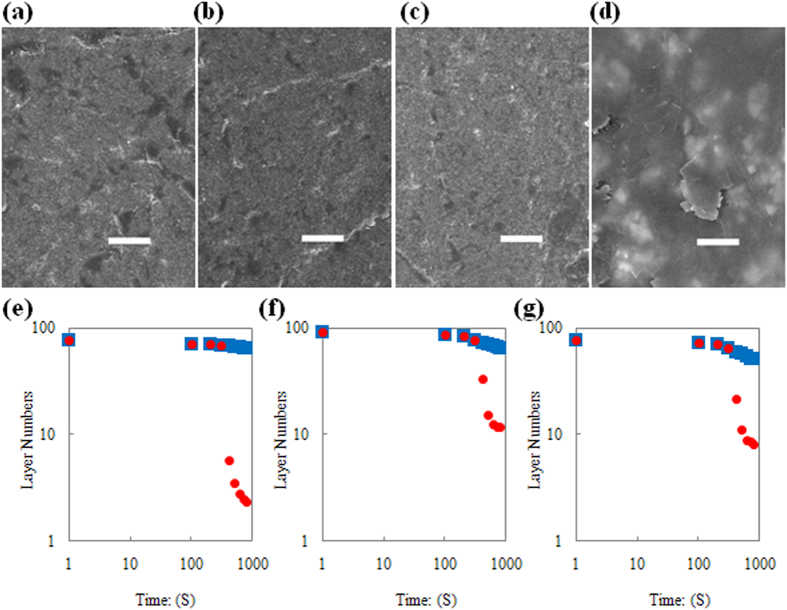
Additional comparative results on mechanisms for layer number controllability. (**a**) SEM image of sandpaper’s surface after 100 seconds Si initial smoothing rubbing (scale bar, 20 μm). (**b**) SEM image of sandpaper’s surface after 200 seconds Si initial smoothing rubbing (scale bar, 20 μm). (**c**) SEM image of sandpaper’s surface after 300 seconds Si initial smoothing rubbing (scale bar, 20 μm). (**d**) SEM image of smooth region on sandpaper’s surface, after 300 seconds Si initial smoothing rubbing (scale bar, 1 μm). (**e**) Statistics results of graphene with certain layer numbers. (**e**) Layer number of graphene on PET substrates as a function of double smoothing rubbing conditions. (**f**) Layer number of graphene on SiO_2_ substrates as a function of double smoothing rubbing conditions. (**g**) Layer number of graphene on Si substrates as a function of double smoothing rubbing conditions.

For Condition 1, the smooth region did not increase significantly as the rubbing time increased (compare Fig. 7a–c) even though the smooth regions were sufficiently smooth (Fig. 7d). Thus, for Condition 1, no mono- or bilayer graphene could be obtained (see the blue points in Fig. 7e–g).

For Condition 2, as described in Fig. 6, the double-smoothing-rubbing step used PET as both the initial and second smoothing material. Although the smooth region became sufficiently large, the smoothness of the smooth region could not be sufficiently improved. Thus, there are considerably more layers in the final samples than in the samples obtained by the procedure shown in Fig. 1.

Similar results were obtained under Condition 3 as under Condition 2 (compare the red points in Fig. 7e–g and the black/blue points in Fig. 3f–h).Si, as the initial smoothing material, could not create a sufficient amount of smooth regions, whereas PET, as the second smoothing material, created larger smooth regions but did not adequately improve the smoothness of the existing smooth regions.

The PET smoothing material after the double-smoothing-rubbing step could be reused directly for the restorative rubbing step without any further processing (Figure S8 present the morphology of the PET smoothing material after the double-smoothing-rubbing step). The same applies for the Si smoothing material used in the double-smoothing-rubbing step; however, a brief ultrasonic cleaning was performed prior to reuse.

**Figure 8:**
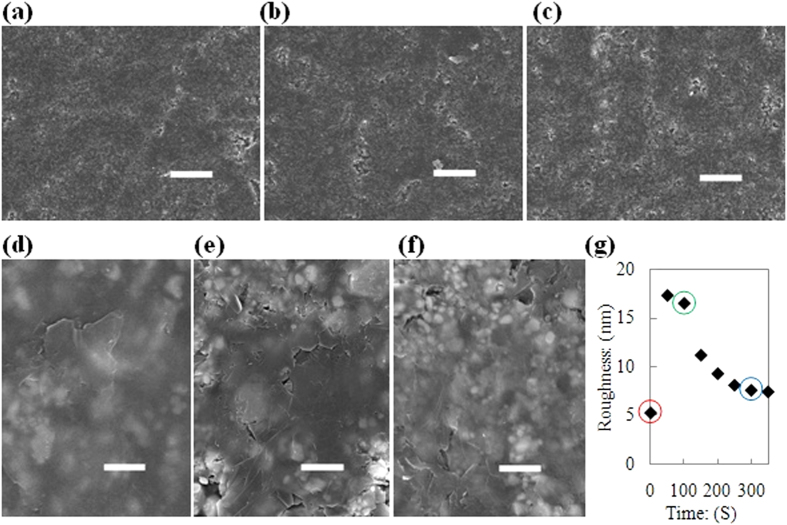
Mechanisms for Restoring Smoothing Rubbing. (**a**) SEM image of sandpaper’s surface after a round of soft contact rubbing (scale bar, 20 μm). (**b**) SEM image of sandpaper’s surface after 100 seconds PET-sandpaper rubbing (scale bar, 20 μm). (**c**) SEM image of sandpaper’s surface after 200 seconds Si smoothing rubbing (scale bar, 20 μm). (**d**) SEM image of smooth region on sandpaper’s surface, after a round of soft contact rubbing (scale bar, 1 μm). (**e**) SEM image of smooth region on sandpaper’s surface, after 100 seconds PET-sandpaper rubbing (scale bar, 1 μm). (**f**) SEM image of smooth region on sandpaper’s surface, after 200 seconds Si smoothing rubbing (scale bar, 1 μm). (**g**) Smoothness of smooth region on sandpaper’s surface as a function of Restoring Smoothing Rubbing process flow. (Point in red cycle: smoothness of smooth region on sandpaper’s surface after a round of soft contact rubbing. Point in green cycle: smoothness of smooth region on sandpaper’s surface after 100 seconds PET-sandpaper rubbing. Point in blue cycle: smoothness of smooth region on sandpaper’s surface after 200 seconds Si smoothing rubbing.)

During the restorative-rubbing step, the ratios of the coarse region to the smooth region did not change significantly (compare Fig. 8a–c). However, the smoothness of the smooth regions was altered considerably (compare Fig. 8d–f) and restored to a favourable smoothness (i.e., restored to a similar smoothness as the sandpaper surface after the double smoothing rubbing step) by a Si smoothing rubbing procedure. Figure 8g shows the change in the roughness of the smooth regions during the restorative rubbing step, which again demonstrates the importance of smooth areas on the sandpaper.

In summary, we achieved a rapid transfer-free fabrication of graphene on different substrates (both rigid and flexible substrates) at room temperature by a rubbing method. This highly efficient and low-cost method used inexpensive commercial graphite as a raw material. Mono- and few-layer graphene films were fabricated directly on various substrates with an average production time of less than one minute. The size of the deposited graphene flakes was several to tens of micrometres. The graphene was characterized as having a high elemental purity, with defects consisting primarily of boundary (or edge) types. The rubbing method displayed good controllability of the graphene-covered area and of the number of graphene layers on the substrates. The graphene films produced by this method performed well in applications, such as humidity sensing, transparent heating, and strain sensing, thus demonstrating the potential industrial and commercial value of the technique. Furthermore, this rubbing method can be extended to other 2-D atomic crystals.

## Methods

### Raw Materials

Poly(ethylene terephthalate) (PET) films (Shenzhen Yichuan Thin Film Co., Ltd.) were needed as initial smoothing material (light gray piece in Fig. 1b). Polished silicon (Si) wafers (Zhejiang Lijing Silicon Materials Co., Ltd.) were needed as further smoothing material (dark blue piece in Fig. 1b). Polished Si wafers, polished quartz (SiO_2_) glasses (Jiangsu Jinghe Optical Instrument Co., Ltd.) and PET films were used as substrates (the substrates are shown in Fig. 1d as light blue pieces, which are marked by “Substrate-1”, “Substrate-2”…… and “Substrate-n”). Polishing sandpapers (12000 mesh, 3M Company) were required as the basement of “Reusable Surfaces” (the sandpapers were shown as light green pieces in Fig. 1a). Three kinds of commercial graphite powders were chosen: graphite powder from Aladdin Industrial Inc., graphite powder from Qingdao Chenyang Graphite Co., Ltd and pyrolytic graphite powder from Nanjing XFNANO Materials Tech Co., Ltd. Furthermore, h-BN (Aladdin Industrial Inc.), MoS_2_ (Aladdin Industrial Inc.) and WS_2_ (Aladdin Industrial Inc.) powders are needed as raw materials for other 2-D atomic crystals.

### Rubbing details

All of the rubbing steps shared the following characteristics: (1) The rubbing action can be described as “revolution without self-rotation”. The rubbing diameter was 0.5 cm, and the rubbing rate was 200 rpm. (2) A commercial rubber (Silicone rubber sheet, Wuhan Great Wall Rubber Co., Ltd.) was needed to apply the appropriate pressures. It was affixed to the rubbing equipment, as shown in Figure S9.

### Sandpaper-rubbing

The commercial raw graphene powder was rubbed between two pieces of polishing sandpaper (Fig. 1a) by applying a gentle pressure (0.1 MPa) for 100 s.

After this rubbing step, the polishing sandpaper surface would be covered by graphite sheets or other 2-D sheets (light green pieces covered by a dark area in Fig. 1b).

### Double-smoothing-rubbing

As shown in Fig. 1b, this step includes two sub-steps. First, the PET-smoothing-rubbing step involved the use of PET as the initial smoothing material. Then, the Si-smoothing-rubbing step utilized a polished Si wafer as the second smoothing material.

Initially, as shown in the left part of Fig. 1b, we rubbed the two pieces of polishing sandpapers (already covered by graphite sheets or other 2-D sheets) on PET films by applying soft pressure (0.04MPa) for different amounts of time (100 to 400s). After this rubbing process, the polishing sandpapers’ surfaces had a ratio of smooth to coarse regions that corresponded to the rubbing time.

As shown in the right part of Fig. 1b, we then rubbed the polishing sandpaper on a polished Si wafer by applying a soft pressure (0.2MPa) for 100 to 400s. After this rubbing process, the polishing sandpaper’s surface had different degrees of smoothness that corresponded to the rubbing time.

### Repeated-production

As shown in Fig. 1c, this step consisted of two steps that could be performed in parallel. One is the soft-contact-rubbing step, and the other is the restorative-rubbing step.

During one round of production, one piece of reusable surface was used for the production of graphene on a certain type of substrate by the soft-contact-rubbing step (marked in the red shadow boxes in Fig. 1c, and shown in detail as Fig. 1d), while the other piece of reusable surface could be used in the restorative-rubbing step (marked in the blue shadow boxes in Fig. 1c, and shown in detail as Fig. 1e).

The two steps are described below.

### Details of soft-contact-rubbing

As shown in Fig. 1d, after contacting a target substrate (Si, SiO_2_ or PET) with a commercial rubber, the target substrate was rubbed with the reusable surface by applying a soft pressure (0.5 MPa for rigid substrates and 0.1 MPa for flexible substrates) for 20 to 40 s. This rubbing process fabricated the final samples (graphene or other 2-D atomic crystals on a substrate).

### Details of restorative-rubbing

As shown in Fig. 1e, this step includes three sub-steps. First, a proper amount of graphite or other 2-D raw powder is rubbed between PET smoothing materials. Second, the PET smoothing material is rubbed against the reusable surface. Third, the reusable surface rubbed against the polished Si wafer.

Initially, a proper amount of graphite or other 2-D raw powder was added and then rubbed between the two PET smoothing materials (the PET smoothing materials used during the double-smoothing-rubbing step can be re-used here) by applying a soft pressure (0.1 MPa) for 100 s.

Then, we rubbed the reusable surface on the PET smooth material by applying a soft pressure (0.1MPa) for 100s. The PET smoothing material can be reused for the next restorative rubbing step. From our experiments, the PET smoothing materials can be reused 20 times.

Finally, we affixed the polished Si wafer (after a one-minute ultrasonic cleaning and drying) to the commercial rubber, and we rubbed the reusable surface on the Si by applying a soft pressure (0.2 MPa) for 200 s. The Si smoothing material could be reused for the next restorative-rubbing step, following the cleaning step described above. From our experiments, the Si smoothing materials can be reused more than 100 times.

After the above three rubbing steps, the reusable surface was ready for another cycle.

### Materials Characterizations

Raman spectra of graphene and other 2-D atomic crystals on Si substrates were characterized by 514 nm Raman spectrometry (INVIA), and the 10 μm × 10 μm area Raman mapping images were obtained from Raman laser whose spot size is 1 μm × 1 μm was moved by 1 μm. The thicknesses of graphene and other 2-D atomic crystals on Si substrates were tested by AFM (DI Nanoscope IV, tapping mode with silicon tips). The edges of monolayer graphene and other 2-D atomic crystals (peeled from SiO_2_ substrates) were characterized by TEM (JEM-2100F) after transferred onto TEM grid, and the elemental purity of the graphene was detected by EDS. The sizes and distribution of 2-D atomic crystals on SiO_2_ substrates were observed by optical microscopy (XSP-35). The morphology of graphene and other 2-D atomic crystals on PET substrates and the morphology changes of polishing sandpapers’ surfaces were characterized by SEM (Sirion 200 and Nova NanoSEM 450). Sheet resistance and optical transparency measurements of flexible transparent conductive films (graphene/SiO_2_ and graphene/PET samples) were taken by Four-probe tester (RTS-8) and UV-Vis Spectra photometry (Lambda 35), respectively.

### Humidity Sensors

A 30 s soft-contact-rubbing was chosen for humidity sensing. The sensors were fabricated by printing silver pads (3 cm × 0.25 cm) onto 3 cm × 3 cm sized graphene/SiO_2_ and graphene/PET in a pre-defined pattern. The humidity sensing behaviours were tested in a constant temperature and humidity incubator (SPX-160B) at a constant temperature of 300 K while the relative humidity was changed from 30% to 90%.

### Flexible transparent heaters

A 40 s soft-contact-rubbing was chosen for transparent heating. The heaters were fabricated by printing silver pads (6 cm × 0.25 cm) onto 6 cm × 6 cm graphene/SiO_2_ and graphene/PET in a pre-defined pattern. A polycarbonate (PC) film was used as an insulating protective layer for security. The alternating current (AC) working voltage was provided by an adjustable regulated AC power supply. The heating temperature was measured using an infrared thermometer.

### Strain Sensors

For strain sensing, a 25 s soft-contact-rubbing was chosen. The sensors were fabricated by printing silver pads (12 cm × 0.25 cm) onto 12 cm × 12 cm sized graphene/PU in a pre-defined pattern. The strain gauge factors were calculated by testing the resistivity changes under strain-on/off conditions. The strain was performed and measured by a strain equipment made by ourselves. In this study, “strain off” means 0% strain (without elongation), and “strain on” means approximately 3% strain (with elongation as approximately 0.36 mm).

## Additional Information

**How to cite this article**: Jiang, S. *et al*. One-Minute Room-Temperature Transfer-Free Production of Mono- and Few-Layer Polycrystalline Graphene on Various Substrates. *Sci. Rep*. **6**, 19313; doi: 10.1038/srep19313 (2016).

## Supplementary Material

Supplementary Information

## References

[b1] Ferrari A. C. . Science and Technology Roadmap for Graphene, Related Two-Dimensional Crystals, and Hybrid Systems. Nanoscale 7, 4598–4810 (2015). 25707682 10.1039/c4nr01600a

[b2] Yazyev O. V. & Chen Y. P. Polycrystalline Graphene and Other Two-Dimensional Materials. Nat. Nanotech. 9, 755–767 (2014). 10.1038/nnano.2014.166 25152238

[b3] Novoselov K. S. . A Roadmap for Graphene. Nature 490, 192–200 (2012). 23060189 10.1038/nature11458

[b4] Ren W. & Cheng H. The Global Growth of Graphene. Nat. Nanotech. 9, 726–730 (2014). 10.1038/nnano.2014.229 25286256

[b5] Paton K. R. . Scalable Production of Large Quantities of Defect-Free Few-Layer Graphene by Shear Exfoliation in Liquids. Nat. Mater. 7, 624–630 (2014). 24747780 10.1038/nmat3944

[b6] Nicolosi V., Chhowalla M., Kanatzidis M. G., Strano M. S. & Coleman J. N. Liquid Exfoliation of Layered Materials. Science 340, 6139–6156 (2013).

[b7] Coleman J. N. Liquid Exfoliation of Defect-Free Graphene. Acc. Chem. Res. 46, 14–22 (2013). 22433117 10.1021/ar300009f

[b8] James D. K. & Tour J. M. Graphene: Powder, Flakes, Ribbons, and Sheets. Acc. Chem. Res. 46, 2307–2318 (2013). 23276286 10.1021/ar300127r

[b9] Zhu Y., James D. K. & Tour J. M. New Routes to Graphene, Graphene Oxide and Their Related Applications. Adv. Mater. 24, 4924–4955 (2012). 22903803 10.1002/adma.201202321

[b10] Bonaccorso F. . Production and Processing of Graphene and 2d Crystals. Mater. Today 15, 564–589 (2012).

[b11] Akinwande D., Petrone N. & Hone J. Two-Dimensional Flexible Nanoelectronics. Nat. Commun. 5, 5678 (2014). 25517105 10.1038/ncomms6678

[b12] Yan Z., Peng Z. & Tour J. M. Chemical Vapor Deposition of Graphene Single Crystals. Acc. Chem. Res. 47, 1327–1337 (2014). 24527957 10.1021/ar4003043

[b13] Du J., Pei S., Ma L. & Cheng H. 25th Anniversary Article: Carbon Nanotube- and Graphene-Based Transparent Conductive Films for Optoelectronic Devices. Adv. Mater. 26, 1958–1991 (2014). 24591083 10.1002/adma.201304135

[b14] Yan K., Fu L., Peng H. & Liu Z. Designed CVD Growth of Graphene via Process Engineering. Acc. Chem. Res. 46, 2263–2274 (2013). 23869401 10.1021/ar400057n

[b15] Zhang Y., Zhang L. & Zhou C. Review of Chemical Vapor Deposition of Graphene and Related Applications. Acc. Chem. Res. 46, 2329–2339 (2013). 23480816 10.1021/ar300203n

[b16] Kang J., Shin D., Bae S. & Hong B. H. Graphene Transfer: Key for Applications. Nanoscale 4, 5527–5537 (2012). 22864991 10.1039/c2nr31317k

[b17] Novoselov K. S. Rapid Progress in Producing Graphene. Nature 505, 291 (2014). 24429617 10.1038/505291c

[b18] Zurutuza A. & Marinelli C. Challenges and Opportunities in Graphene Commercialization. Nat. Nanotechnol. 9, 730–734 (2014). 25286257 10.1038/nnano.2014.225

[b19] Peplow M. The Quest for Supercarbon. Nature 503, 327–329 (2013). 24256790 10.1038/503327a

[b20] Kyle J. R., Ozkan C. S. & Ozkan M. Industrial Graphene Metrology. Nanoscale 4, 3807–3819 (2012). 22538861 10.1039/c2nr30093a

[b21] Dahal A. & Batzill M. Graphene–Nickel Interfaces: a Review. Nanoscale 6, 2548–2562 (2012). 24477601 10.1039/c3nr05279f

[b22] Zhuo Q. . Transfer-Free Synthesis of Doped and Patterned Graphene Films. ACS Nano 9, 594–601 (2015). 25544387 10.1021/nn505913v

[b23] McNerny D. Q. . Direct Fabrication of Graphene on SiO_2_ Enabled by Thin Film Stress Engineering. Sci. Rep. 4, 5049 (2014). 24854632 10.1038/srep05049 PMC4031480

[b24] Kim H. . Copper-Vapor-Assisted Chemical Vapor Deposition for High-Quality and Metal-Free Single-Layer Graphene on Amorphous SiO_2_ Substrate. ACS Nano 7, 6575–6582 (2013). 23869700 10.1021/nn402847w

[b25] Teng P. . Remote Catalyzation for Direct Formation of Graphene Layers on Oxides. Nano Lett. 12, 1379–1384 (2012). 22332771 10.1021/nl204024k

[b26] Su C. . Direct Formation of Wafer Scale Graphene Thin Layers on Insulating Substrates by Chemical Vapor Deposition. Nano Lett. 11, 3612–3616 (2011). 21834558 10.1021/nl201362n

[b27] Tang S. . Silane-Catalysed Fast Growth of Large Single-Crystalline Graphene on Hexagonal Boron Nitride. Nat. Commun. 6, 6499 (2015). 25757864 10.1038/ncomms7499 PMC4382696

[b28] Chen J. . Near-Equilibrium Chemical Vapor Deposition of High-Quality Single-Crystal Graphene Directly on Various Dielectric Substrates. Adv. Mater. 26, 1348–1353 (2014). 24338972 10.1002/adma.201304872

[b29] Sun J. . Direct Growth of High-Quality Graphene on High-κ Dielectric SrTiO_3_ Substrates. J. Am. Chem. Soc. 136, 6574–6577 (2014). 24746139 10.1021/ja5022602

[b30] Kim Y. . Direct Integration of Polycrystalline Graphene into Light Emitting Diodes by Plasma-Assisted Metal-Catalyst-Free Synthesis. ACS Nano 8, 2230–2236 (2014). 24506543 10.1021/nn405477f

[b31] Chen J. . Two-Stage Metal-Catalyst-Free Growth of High-Quality Polycrystalline Graphene Films on Silicon Nitride Substrates. Adv. Mater. 25, 992–997 (2013). 23161470 10.1002/adma.201202973

[b32] Wang M. . A Platform for Large-Scale Graphene Electronics – CVD Growth of Single-Layer Graphene on CVD-Grown Hexagonal Boron Nitride. Adv. Mater. 25, 2746–2752 (2013). 23576235 10.1002/adma.201204904

[b33] Wang G. . Direct Growth of Graphene Film on Germanium Substrate. Sci. Rep. 3, 2465 (2013). 23955352 10.1038/srep02465 PMC3746207

[b34] Tang S. . Precisely Aligned Graphene Grown on Hexagonal Boron Nitride by Catalyst Free Chemical Vapor Deposition. Sci. Rep. 2013, *3*, 2666. 10.1038/srep02666 PMC3773621 24036628

[b35] Medina H. . Metal-Free Growth of Nanographene on Silicon Oxides for Transparent Conducting Applications. Adv. Funct. Mater. 22, 2123–2128 (2012).

[b36] Fanton M. A. . Characterization of Graphene Films and Transistors Grown on Sapphire by Metal-Free Chemical Vapor Deposition. ACS Nano 5, 8062–8069 (2011). 21905713 10.1021/nn202643t

[b37] Xiong W. . Single-Step Formation of Graphene on Dielectric Surfaces. Adv. Mater. 25, 630–634 (2013). 23136061 10.1002/adma.201202840

[b38] Kwak J. . Near Room-Temperature Synthesis of Transfer-Free Graphene Films. Nat. Commun. 3, 645 (2012). 22273683 10.1038/ncomms1650

[b39] Kato T. & Hatakeyama R. Direct Growth of Doping-Density-Controlled Hexagonal Graphene on SiO_2_ Substrate by Rapid-Heating Plasma CVD. ACS Nano 6, 8508–8515 (2012). 22971147 10.1021/nn302290z

[b40] Shin H. . Transfer-Free Growth of Few-Layer Graphene by Self-Assembled Monolayers. Adv. Mater. 23, 4392–4397 (2011). 21882264 10.1002/adma.201102526

[b41] Pang Z., Yan Z., Sun Z. & Tour J. M. Direct Growth of Bilayer Graphene on SiO_2_ Substrates by Carbon Diffusion through Nickel. ACS Nano 5, 8241–8247 (2011). 21888426 10.1021/nn202923y

[b42] Cummings A. W. . Charge Transport in Polycrystalline Graphene: Challenges and Opportunities. Adv. Mater. 26, 5079–5094 (2014). 24903153 10.1002/adma.201401389

[b43] Tsen A. W., Brown L., Havener R. W. & Park J. Polycrystallinity and Stacking in CVD Graphene. Acc. Chem. Res. 46, 2286–2296 (2013). 23135386 10.1021/ar300190z

[b44] Butler S. Z. . Progress, Challenges, and Opportunities in Two-Dimensional Materials Beyond Graphene. ACS Nano 7, 2898–2926 (2013). 23464873 10.1021/nn400280c

[b45] C.-Gomez A. . Deterministic Transfer of Two-Dimensional Materials by All-Dry Viscoelastic Stamping. 2D Mater. 1, 011002 (2014).

[b46] Yu Y. . Surface Fractal Evolution Induced Rubbing for Rapid Room Temperature and Transfer-Free Fabrication of Graphene on Flexible Polymer Substrate. Appl. Phys. Lett. 103, 011601 (2013).

[b47] Yu Y. . Room Temperature Rubbing for Few-Layer Two-Dimensional Thin Flakes Directly on Flexible Polymer Substrates. Sci. Rep. 3, 2697 (2013). 24045289 10.1038/srep02697 PMC3776208

[b48] Rafiee J. . Wetting Transparency of Graphene. Nat. Mater. 11, 217–222 (2012). 22266468 10.1038/nmat3228

[b49] Eckmann A. . Probing the Nature of Defects in Graphene by Raman Spectroscopy. Nano Lett. 12, 3925–3930 (2012). 22764888 10.1021/nl300901a

[b50] Batzill M. The Surface Science of Graphene: Metal Interfaces, CVD Synthesis, Nanoribbons, Chemical Modifications, and Defects. Surf. Sci. Rep. 67, 83–115 (2012).

[b51] Zandiatashbar A. . Effect of Defects on the Intrinsic Strength and Stiffness of Graphene. Nat. Commun. 5, 3186 (2014). 24458268 10.1038/ncomms4186

[b52] Zhang Z., Yang Y., Xu F., Wang L. & Yakobson B. I. Unraveling the Sinuous Grain Boundaries in Graphene. Adv. Funct. Mater. 25, 367–373 (2015).

[b53] Hempel M., Nezich D., Kong J. & Hofmann M. A Novel Class of Strain Gauges Based on Layered Percolative Films of 2D Materials. Nano Lett. 12, 5714–5718 (2012). 23045955 10.1021/nl302959a

[b54] Zou X. & Yakobson B. I. An Open Canvas – 2D Materials with Defects, Disorder, and Functionality. Acc. Chem. Res. 48, 73–80 (2015). 25514190 10.1021/ar500302q

[b55] Lv R. . Transition Metal Dichalcogenides and Beyond: Synthesis, Properties, and Applications of Single- and Few-Layer Nanosheets. Acc. Chem. Res. 48, 56–64 (2015). 25490673 10.1021/ar5002846

[b56] George A. S. . Wafer Scale Synthesis and High Resolution Structural Characterization of Atomically Thin MoS_2_ Layers. Adv. Funct. Mater. 24, 7461–7466 (2014).

[b57] P.-López N. . CVD-Grown Monolayered MoS_2_ as an Effective Photosensor Operating at Low-Voltage. 2D Mater. 1, 011004 (2014).

[b58] Bhushan B. Introduction to Tribology (Second Edition), 319–340 (John Wiley & Sons, 2013).

[b59] Straffelini G. Friction and Wear-Methodologies for Design and Control, 86–89 (Springer, 2015).

[b60] Berman D., Erdemir A. & Sumant A. V. Graphene: a New Emerging Lubricant. Mater. Today 17, 31–42 (2014).

