# Supplementary material for: One-Minute Room-Temperature Transfer-Free Production of Mono- and Few-Layer Polycrystalline Graphene on Various Substrates
Source: Sci Rep. 2016 Jan 14;6:19313. doi: 10.1038/srep19313 (PMC4725863; doi:10.1038/srep19313)
Supplement: Supplementary Information [file srep19313-s1.doc]

**Supplementary information**

**One-Minute Room-Temperature Transfer-Free Production of Mono- and Few-Layer Polycrystalline Graphene on Various Substrates**

Shenglin Jiang, Yike Zeng, Wenli Zhou, Xiangshui Miao, Yan Yu


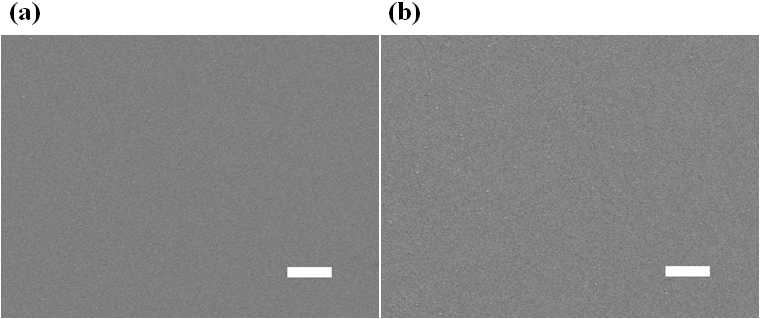


**Figure S1** | Surface SEM images of (a) PET film (scale bar, 5 μm), and (b) polished Silicon wafer (scale bar, 5 μm).


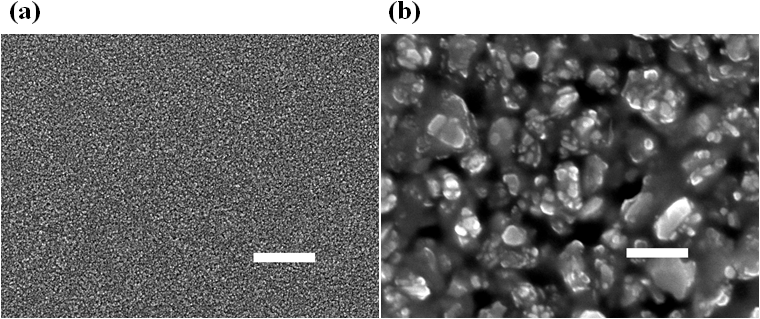


**Figure S2** | Surface SEM images of polished sandpaper with different magnifications, (a) low magnification image (scale bar, 20 μm), and (b) high magnification image (scale bar, 1 μm).


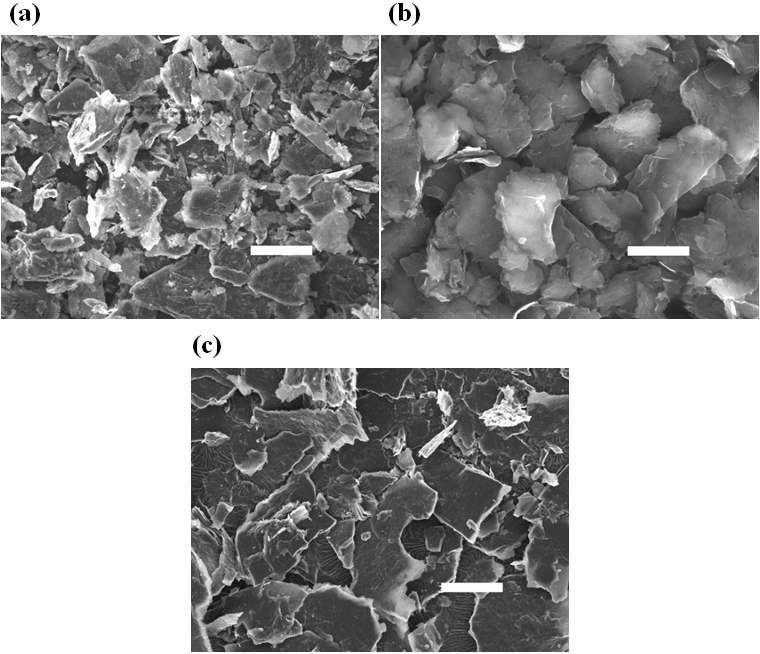


**Figure S3** | SEM images of different graphite powders, (a) graphite from Aladdin Industrial Inc. (scale bar, 5 μm), (b) graphite from Qingdao Chenyang Graphite Co., Ltd (scale bar, 5 μm), and (c) pyrolytic graphite from Nanjing XFNANO Materials Tech Co., Ltd (scale bar, 5 μm).


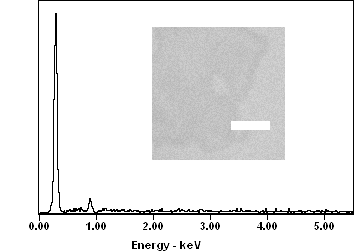


Intensity

**Figure S4** | TEM and corresponding EDS images of graphene (Scale bar: 100 nm).


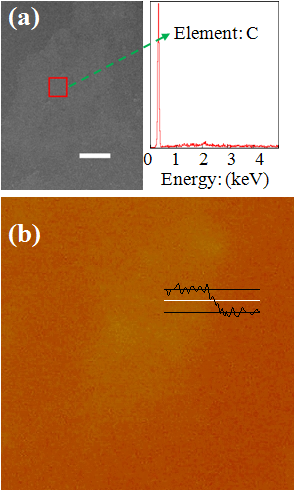


1.21 nm

**Figure S5** | (a) SEM image and energy dispersive spectroscopy (EDS) results of graphene on PE substrate (scale bar, 1 μm). (b) AFM image of a liquid exfoliated graphene flake deposited on Si substrate (area: 10 μm × 10 μm).

**Layer number estimation method from AFM results.**

According to literature reports (references S2–S5), the layer number of two-dimensional flakes can be calculated as the following equation.

*N*=*(d*－*d_0_)*/*△d*

In this equation, *d* is the tested results by AFM while *d_0_* is the thickness of mono-layer flake on substrate, and *△d* is the distance between each layer.

For example, for graphene and graphite flakes, *△d* is 0.34 nm. And according to literature results (references S5–S9), graphene monolayers on substrates are approximately 0.65 ~ 0.95 nm thick. Thus *d_0_* for graphene and graphite flakes is ranged from 0.65 ~ 0.95.

**Table S1** | Error data for the black points in Figure 3f.

| Time Length | 50 | 100 | 150 | 200 | 250 | 300 |
| --- | --- | --- | --- | --- | --- | --- |
| Average | 10.4 | 6.2 | 4.7 | 3.9 | 3.1 | 2.7 |
| Highest | 23 | 14 | 11 | 9 | 7 | 6 |
| Lowest | 6 | 4 | 3 | 2 | 2 | 2 |

**Table S2** | Error data for the blue points in Figure 3f.

| Time Length | 300 | 400 | 500 | 600 | 700 | 800 |
| --- | --- | --- | --- | --- | --- | --- |
| Average | 2.7 | 2.5 | 2.3 | 2.2 | 2.2 | 2.1 |
| Highest | 6 | 6 | 6 | 4 | 4 | 5 |
| Lowest | 2 | 2 | 1 | 2 | 2 | 1 |

**Table S3** | Error data for the red points in Figure 3f.

| Time Length | 300 | 400 | 500 | 600 | 700 | 800 |
| --- | --- | --- | --- | --- | --- | --- |
| Average | 2.7 | 2.0 | 1.5 | 1.4 | 1.4 | 1.3 |
| Highest | 5 | 5 | 3 | 4 | 4 | 3 |
| Lowest | 1 | 1 | 1 | 1 | 1 | 1 |

**Table S4** | Error data for the black points in Figure 3g.

| Time Length | 50 | 100 | 150 | 200 | 250 | 300 |
| --- | --- | --- | --- | --- | --- | --- |
| Average | 53.4 | 29.1 | 19.2 | 15.4 | 13.3 | 12.6 |
| Highest | 72 | 47 | 32 | 22 | 19 | 17 |
| Lowest | 43 | 17 | 13 | 11 | 9 | 8 |

**Table S5** | Error data for the blue points in Figure 3g.

| Time Length | 300 | 400 | 500 | 600 | 700 | 800 |
| --- | --- | --- | --- | --- | --- | --- |
| Average | 12.6 | 12.3 | 12.1 | 11.9 | 11.8 | 11.6 |
| Highest | 19 | 19 | 17 | 17 | 18 | 16 |
| Lowest | 9 | 9 | 9 | 7 | 7 | 8 |

**Table S6** | Error data for the red points in Figure 3g.

| Time Length | 300 | 400 | 500 | 600 | 700 | 800 |
| --- | --- | --- | --- | --- | --- | --- |
| Average | 12.6 | 4.6 | 2.2 | 1.8 | 1.7 | 1.6 |
| Highest | 19 | 9 | 5 | 4 | 4 | 4 |
| Lowest | 9 | 2 | 1 | 1 | 1 | 1 |

**Table S7** | Error data for the black points in Figure 3h.

| Time Length | 50 | 100 | 150 | 200 | 250 | 300 |
| --- | --- | --- | --- | --- | --- | --- |
| Average | 43.1 | 25.5 | 17.4 | 13.2 | 10.9 | 9.5 |
| Highest | 66 | 41 | 28 | 19 | 17 | 16 |
| Lowest | 29 | 16 | 12 | 10 | 9 | 6 |

**Table S8** | Error data for the blue points in Figure 3h.

| Time Length | 300 | 400 | 500 | 600 | 700 | 800 |
| --- | --- | --- | --- | --- | --- | --- |
| Average | 9.5 | 9.2 | 8.9 | 8.7 | 8.6 | 8.5 |
| Highest | 16 | 16 | 15 | 15 | 14 | 15 |
| Lowest | 6 | 6 | 6 | 4 | 5 | 5 |

**Table S9** | Error data for the red points in Figure 3h.

| Time Length | 300 | 400 | 500 | 600 | 700 | 800 |
| --- | --- | --- | --- | --- | --- | --- |
| Average | 9.5 | 3.7 | 2.1 | 1.6 | 1.5 | 1.5 |
| Highest | 16 | 7 | 4 | 4 | 4 | 4 |
| Lowest | 6 | 2 | 2 | 1 | 1 | 1 |


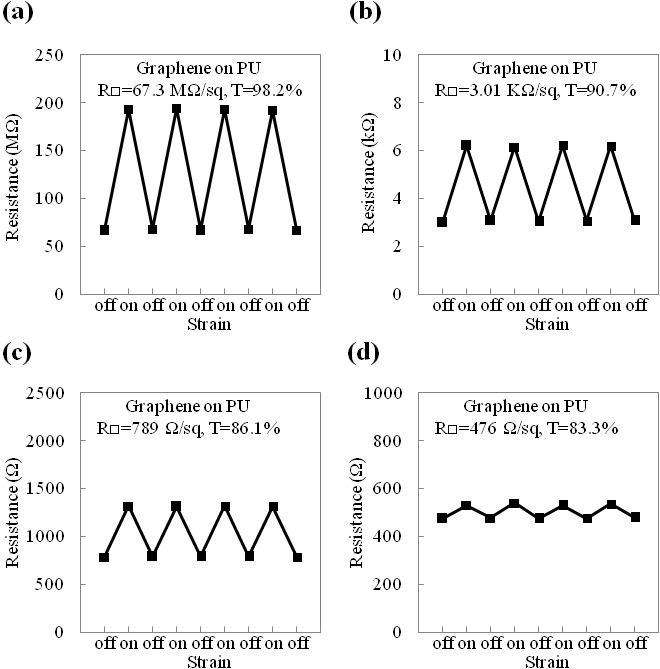


**Figure S6** | Strain sensing property of graphene/PU samples (with different soft-contact-rubbing time): (a) 20 s (strain gauge factor is 61). (b) 30 s (strain gauge factor is 34). (c) 35 s (strain gauge factor is 22). (4) 40 s (strain gauge factor is 4).


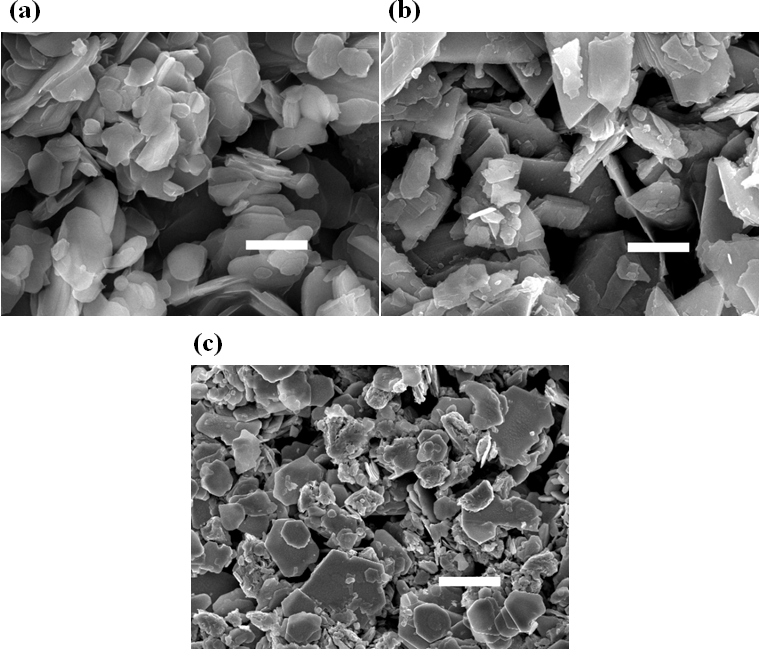


**Figure S7** | SEM images of different 2-D raw materials powders, (a) h-BN from Aladdin Industrial Inc. (scale bar, 5 μm), (b) MoS_2_ from Aladdin Industrial Inc. (scale bar, 5 μm), and (c) WS_2_ from Aladdin Industrial Inc. (scale bar, 5 μm).


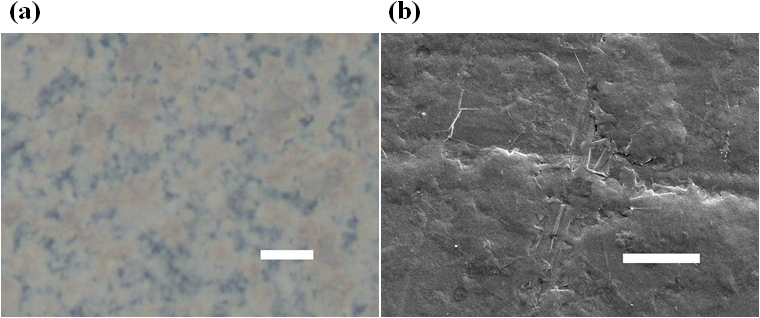


**Figure S8** | Optical and SEM images of PET smoothing material after the Double-Smoothing-Rubbing step, (a) optical image (scale bar, 20 μm), and (b) SEM image (scale bar, 5 μm).


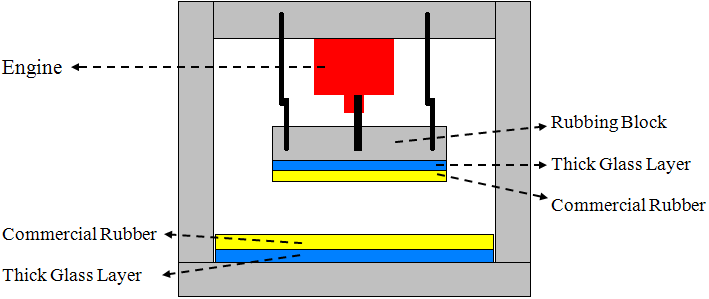


**Figure S9** | Schematic diagram of the rubbing equipment.

**Table S10** | Square Resistivity and Optical Transparency (Wavelength at 550 nm) of Samples with Different Sizes

| Sample Type | 3 cm×3 cm | 6 cm×6 cm | 9 cm×9 cm | 12 cm×12 cm |
| --- | --- | --- | --- | --- |
| Graphene on PET for Humidity Sensing  （10 Samples for Each Size） | Average  R_□_=3.47 KΩ/sq  Average  T=91.1% | Average  R_□_=3.32 KΩ/sq  Average  T=91.6% | Average  R_□_=3.50 KΩ/sq  Average  T=92.2% | Average  R_□_=3.44 KΩ/sq  Average  T=91.1% |
| Graphene on SiO_2_ for Humidity Sensing  （10 Samples for Each Size） | Average  R_□_=3.36 KΩ/sq  Average  T=92.3% | Average  R_□_=3.39 KΩ/sq  Average  T=92.5% | Average  R_□_=3.31 KΩ/sq  Average  T=92.3% | Average  R_□_=3.42 KΩ/sq  Average  T=92.7% |
| Graphene on PET for Transparent Heaters  （10 Samples for Each Size） | Average  R_□_=557 Ω/sq  Average  T=85.9% | Average  R_□_=563 Ω/sq  Average  T=86.2% | Average  R_□_=569 Ω/sq  Average  T=86.3% | Average  R_□_=550 Ω/sq  Average  T=85.8% |
| Graphene on SiO_2_ for Transparent Heaters  （10 Samples for Each Size） | Average  R_□_=566 Ω/sq  Average  T=86.8% | Average  R_□_=559 Ω/sq  Average  T=86.3% | Average  R_□_=562 Ω/sq  Average  T=86.6% | Average  R_□_=573 Ω/sq  Average  T=87.1% |
| Graphene on PU for Strain Sensing  （20 Samples for Each Size） | Average  R_□_=67.5 MΩ/sq  Average  T=98.1% | Average  R_□_=69.6 MΩ/sq  Average  T=98.2% | Average  R_□_=66.2MΩ/sq  Average  T=97.7% | Average  R_□_=68.1 MΩ/sq  Average  T=97.8% |

**Supplementary references**

1. Kim, J., et al. Direct exfoliation and dispersion of two-dimensional materials in pure water via temperature control. *Nat. Commun.* **6**, 8294 (2015).
2. Hernandez, Y., et al. High-yield production of graphene by liquid-phase exfoliation of graphite. *Nat. Nanotech.* **3**, 563–568 (2008).
3. Zheng, J., et al. High yield exfoliation of two-dimensional chalcogenides using sodium naphthalenide. *Nat. Commun.* **5**, 2995 (2014).
4. Kang, J., Seo, J. T., Alducin, D., Ponce, A., Yacaman, M. J., Hersam, M. C. Thickness sorting of two-dimensional transition metal dichalcogenides via copolymer-assisted density gradient ultracentrifugation. *Nat. Commun.* **5**, 5478 (2014).
5. Paton, K. R., et al. Scalable Production of Large Quantities of Defect-Free Few-Layer Graphene by Shear Exfoliation in Liquids. *Nat. Mater.* **7**, 624–630 (2014).
6. Chen, J., et al. Near-equilibrium chemical vapor deposition of high-quality single-crystal graphene directly on various dielectric substrates. *Adv. Mater.* **26**, 1348–1353 (2014).
7. Kim, Y., et al. Direct Integration of Polycrystalline Graphene into Light Emitting Diodes by Plasma-Assisted Metal-Catalyst-Free Synthesis. *ACS Nano* **8**, 2230–2236 (2014).
8. Wang, G., et al. Direct growth of graphene film on germanium substrate. *Sci. Rep.* **3**, 2465 (2013).
9. Hao, Y. *et al*. Probing layer number and stacking order of few-layer graphene by raman spectroscopy. *Small* **6**, 195–200 (2010).
